# Prognostic value of early sustained ventricular arrhythmias in ST-segment elevation myocardial infarction treated by primary percutaneous coronary intervention: A substudy of VALIDATE-SWEDEHEART trial

**DOI:** 10.1016/j.hroo.2022.12.008

**Published:** 2022-12-22

**Authors:** Marina M. Demidova, Rebecca Rylance, Sasha Koul, Christian Dworeck, Stefan James, Mikael Aasa, Mehmet Hamid, Eva Swahn, Kristina Hambraeus, Mikael Danielewicz, Rikard Linder, Ole Fröbert, Per Grimfjärd, Jason Stewart, Loghman Henareh, Jonas Andersson, Henrik Wagner, David Erlinge, Pyotr G. Platonov

**Affiliations:** ∗ Department of Cardiology, Clinical Sciences, Lund University, Lund, Sweden; † Department of Cardiology, Sahlgrenska University Hospital, Göteborg, Sweden; ‡ Department of Medical Sciences, Uppsala University, Uppsala, Sweden; § Department of Cardiology, Södersjukhuset, Stockholm, Sweden; ¶ Department of Cardiology, Mälarsjukhuset, Eskilstuna, Sweden; || Department of Cardiology, Linköping University Hospital, Linköping, Sweden; ∗∗ Department of Cardiology, Falun Hospital, Falun, Sweden; †† PCI-Unit, Karlstad Hospital, Karlstad, Sweden; ‡‡ Department of Cardiology, Danderyd Hospital, Stockholm, Sweden; §§ Department of Cardiology, Faculty of Health, Örebro University, Örebro, Sweden; ¶¶ Department of Internal Medicine, Västmanlands Hospital, Västerås, Sweden; |||| Department of Cardiology, Skaraborgs Hospital, Skövde, Sweden; ∗∗∗ Department of Cardiology, Karolinska University Hospital, Stockholm, Sweden; ††† Department of Cardiology, Umeå University, Umeå, Sweden; ‡‡‡ Department of Cardiology, Helsingborg Hospital, Helsingborg, Sweden

**Keywords:** STEMI, Ventricular arrhythmias, Monomorphic ventricular tachycardia, Ventricular fibrillation, PCI

## Abstract

**Background:**

Prognostic assessment of ventricular tachycardia (VT) or ventricular fibrillation (VF) in ST-segment elevation myocardial infarction (STEMI) is based mainly on distinguishing between early (<48 hours) and late arrhythmias, and does not take into account its time distribution with regard to reperfusion, or type of arrhythmia.

**Objective:**

We analyzed the prognostic value of early ventricular arrhythmias (VAs) in STEMI with regard to their type and timing.

**Methods:**

The prespecified analysis of the multicenter prospective Bivalirudin versus Heparin in ST-Segment and Non-ST-Segment Elevation Myocardial Infarctionin Patients on Modern Antiplatelet Therapy in the Swedish Web System for Enhancement and Development of Evidence-based Care in Heart Disease evaluated according to Recommended Therapies Registry Trial included 2886 STEMI patients undergoing primary percutaneous coronary intervention (PCI). VA episodes were characterized regarding their type and timing. Survival status at 180 days was assessed through the population registry.

**Results:**

Nonmonomorphic VT or VF was observed in 97 (3.4%) and monomorphic VT in 16 (0.5%) patients. Only 3 (2.7%) early VA episodes occurred after 24 hours from symptom onset. VA was associated with higher risk of death (hazard ratio 3.59; 95% confidence interval [CI] 2.01–6.42) after adjustment for age, sex, and STEMI localization. VA after PCI was associated with an increased mortality compared with VA before PCI (hazard ratio 6.68; 95% CI 2.90–15.41). Early VA was associated with in-hospital mortality (odds ratio 7.39; 95% CI 3.68–14.83) but not with long-term prognosis in patients discharged alive. The type of VA was not associated with mortality.

**Conclusion:**

VA after PCI was associated with an increased mortality compared with VA before PCI. Long-term prognosis did not differ between patients with monomorphic VT and nonmonomorphic VT or VF, but events were few. VA incidence during 24 to 48 hours of STEMI is negligibly low, thus precluding assessment of its prognostic importance.

**Key Findings**

▪ Early ventricular arrythmia (VA) is a marker of poor short-term outcomes in patients with ST-segment elevation myocardial infarction, which does not affect long-term prognosis in patients successfully resuscitated and discharged from hospital.
▪ The incidence of VA during the second day of ST-segment elevation myocardial infarction is negligibly low, supporting the use of a 24-hour, rather than 48-hour, threshold defining early VA not affecting long-term prognosis.
▪ VA that occurs after percutaneous coronary intervention is associated with an increased risk of death compared with VA before percutaneous coronary intervention.

## Introduction

The last several decades demonstrated marked progress in ST-segment elevation myocardial infarction (STEMI) treatment, improving outcomes with early aggressive revascularization and adjunctive antithrombotic treatment. Declining incidence rates and case-fatality rates of ventricular tachycardia (VT) and ventricular fibrillation (VF) in STEMI have been observed in community-based settings.^1^ Nevertheless, malignant ventricular arrhythmias (VAs) still contribute to a 4- to 5-fold increase in in-hospital mortality during STEMI^2^^,^^3^ and remain the main cause of sudden death during the prehospital stage.^4^ In patients successfully resuscitated and discharged from the hospital, early VAs do not appear to influence long-term prognosis.^3^^,^^5^^,^^6^

Prognostic assessment of VAs in STEMI is currently based primarily on categorization of the arrhythmias on early and late based on a 48-hour cutoff time from symptom onset. This cutoff is used to distinguish early VA that do not bear prognostic significance, and late VA that may trigger implantation of an implantable cardioverter-defibrillator. This threshold, however, was set at a time when revascularization using percutaneous coronary intervention (PCI) was not widely implemented as the primary management strategy for STEMI.^7^^,^^8^ Whether different and possibly shorter cutoff times should be considered for patients undergoing invasive strategy is not known. Moreover, time distribution of VAs during STEMI is more complex than the binary division into early and late arrhythmias. It is plausible to suggest that the prognostic importance of prehospital VAs may not be the same as the importance of VAs occurring during reperfusion or after PCI; however, data on prognostic significance of VA occurring prior to, during, and after PCI are scarce and controversial.^5^^,^^9^, ^10^, ^11^

Until recently, prognostic assessment of VAs did not take into account the type of VA.^5^^,^^7^^,^^9^^,^^12^ Recent data support a differential approach to polymorphic VT or VF (PMVT/VF) and monomorphic VT (MMVT), and indicate a long-term mortality hazard associated with MMVT.^13^, ^14^, ^15^

The Bivalirudin versus Heparin in ST-Segment and Non-ST-Segment Elevation Myocardial Infarctionin Patients on Modern Antiplatelet Therapy in the Swedish Web System for Enhancement and Development of Evidence-based Care in Heart Disease evaluated according to Recommended Therapies Registry Trial (VALIDATE-SWEDEHEART) was conducted in contemporary settings with a high adherence to guideline-recommended therapies. This was a multicenter, prospective, randomized, registry-based clinical trial in which VA reporting was integrated.^16^ The design of the VALIDATE-SWEDEHEART trial allows one to overcome the limitations of retrospective studies and prospective studies with selection bias, and based on VALIDATE-VF, we wanted to evaluate the insufficiently elucidated points concerning VAs in STEMI.

The aim of this prespecified analysis of the multicenter prospective VALIDATE-SWEDEHEART trial was to assess the prognostic value of VAs concerning their type and their timing in STEMI patients treated by primary PCI.

## Methods

### Study population

The VALIDATE-SWEDEHEART trial was a multicenter, prospective, randomized, registry-based, controlled, open-label clinical trial aimed at comparing 2 anticoagulation strategies in patients with STEMI or non-STEMI treated by primary PCI. The design and the main results have been published previously.^16^^,^^17^ In brief, patients treated with potent P2Y_12_ inhibitors (ticagrelor, prasugrel, or cangrelor) before start of the intervention were randomized to receive bivalirudin or heparin during PCI, which was performed predominantly using radial artery access. The primary endpoint was a composite of death from any cause, myocardial infarction (MI), and major bleeding events at 180 days. The trial showed that in patients with acute coronary syndromes undergoing primary PCI, bivalirudin was not superior to heparin with regard to the rate of death, repeat MI, or major bleeding events.

The VALIDATE-VF substudy included STEMI patients who were enrolled in the VALIDATE-SWEDEHEART trial. This cohort has been already described in prespecified subgroup analysis.^18^ Baseline patient demographic data, angiographic data, information about medications, and clinical complications were obtained from the SWEDEHEART registry (a nationwide Swedish online registry that reports outcomes of patients hospitalized for acute coronary syndrome or undergoing coronary intervention in Swedish PCI centers). Information on death was obtained from the Swedish National Population Registry.

Information regarding occurrence of arrhythmias requiring medical intervention during index hospitalization with STEMI was collected in accordance with the core protocol of the VALIDATE study. All patients with registered arrhythmias were selected for source data verification in order to then identify patients who developed hemodynamically unstable, sustained VAs during hospitalization for STEMI, and to obtain information on the arrhythmias’ type and timing. With regard to the type of arrhythmia, all cases of documented arrhythmias during STEMI were classified as follows: (1) no arrhythmia, (2) VF or PMVT, (3) MMVT, (4) undefined shockable rhythm, or (5) other nonshockable rhythm requiring cardiopulmonary resuscitation. MMVT was defined as a regular wide QRS complex tachycardia at a rate >100 beats/min with uniform and stable morphology. PMVT was defined as VT with changing QRS morphology. Only hemodynamically unstable sustained VT or VF were selected for further analysis.

Timing of arrhythmia was classified as follows: (1) prior to hospital admission; (2) in hospital before PCI; (3) reperfusion arrhythmia; (4) after PCI, but within 24 hours of symptom onset; (5) after PCI within 24 to 48 hours of symptom onset; and (6) after PCI and later than 48 hours from symptom onset, but before discharge from the hospital. Early VAs were defined as VAs occurring at any time within the first 48 hours of STEMI.^7^

The research reported in this paper was conducted in accordance with the guidelines outlined in the Declaration of Helsinki. Witnessed oral consent was obtained in all patients followed by providing written informed consent within 24 hours.

The study was approved by the ethics committee at Lund University.

### Statistical analysis

Baseline clinical characteristics were presented in comparison between any VA and no VA using Student’s *t* test or the Mann-Whitney test for continuous variables and either the chi-square test or Fisher’s exact test for categorical variables. Adjusted Cox proportional hazards models were performed to evaluate the association between the presence, type, and timing of VAs with the outcome at 180 days. The primary endpoint was total mortality, the secondary endpoint was cardiovascular mortality.

All models were adjusted for age, sex, and localization of STEMI. All tests were 2-sided, and *P* values <.05 were considered significant.

## Results

### Patient characteristics

Between June 2014 and September 2016, a total of 3005 STEMI patients were enrolled in the VALIDATE trial. Among 25 sites that participated in the VALIDATE trial, 16 sites agreed to participate in VALIDATE-VF substudy. In total, 2886 patients were included in the VALIDATE-VF substudy. Of those, 97 (3.4%) experienced VF or PMVT, 16 (0.5%) experienced MMVT, 7 (0.2%) had other undefined shockable rhythm, and 31 (1.1%) had nonshockable rhythm requiring cardiopulmonary resuscitation.

**Figure 1:**
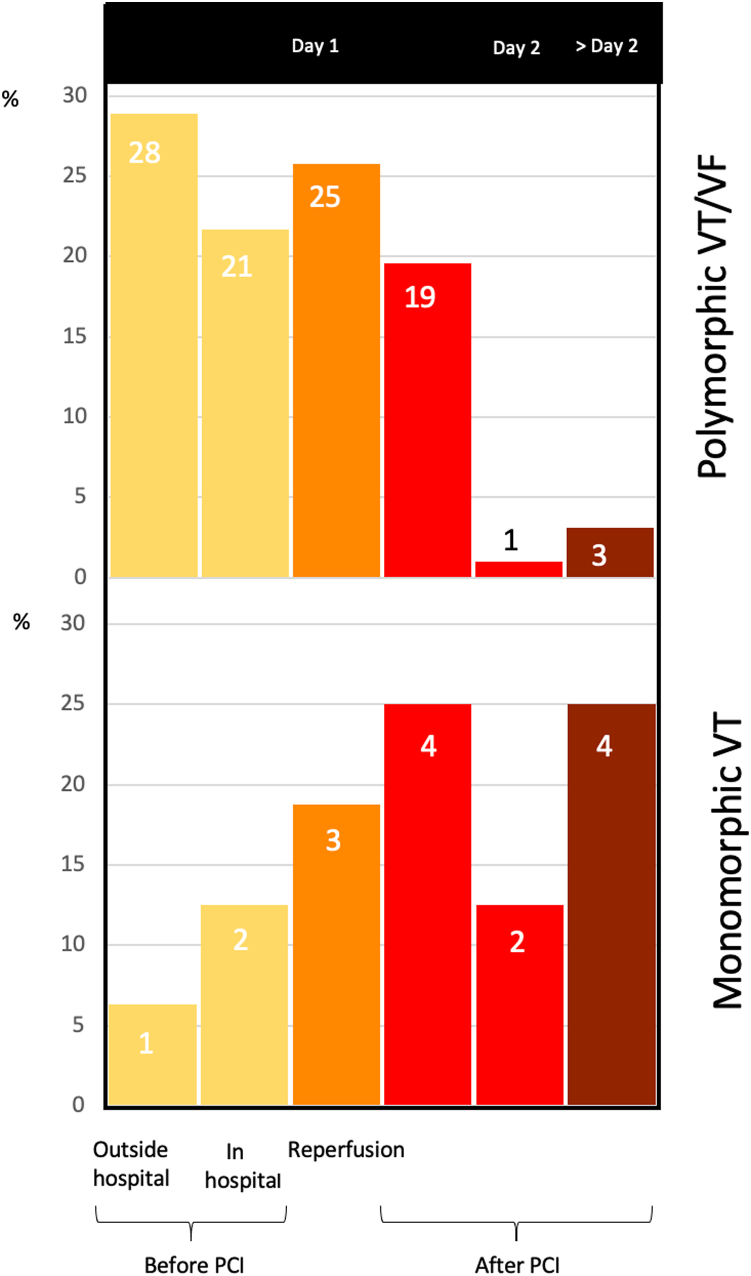
Timing of ventricular tachycardia or ventricular fibrillation (VT/VF) during acute ST-segment elevation myocardial infarction. Data are presented as the percentage of events in each subgroup. Figures in columns indicate the absolute numbers of events in each subgroup. PCI = percutaneous coronary intervention.

In 108 (90%) patients with life-threatening VAs, VT, VF, or shockable rhythm manifested during the first 24 hours of STEMI, in 3 (2.5%) it manifested during the 24 to 48 hours, and in 9 (7.5%) it manifested after 48 hours. Of the total 120 patients with VT, VF, or shockable rhythm, it occurred before reperfusion in 54 (45.4%) patients and directly after reperfusion in 28 (23.5%) patients, and it occurred after PCI, but during hospital stay in 38 (31%) patients (in 29 patients within the first 48 hours of STEMI, and in 8 patients after the 48-hour cutoff). PMVT/VF was most prevalent before PCI, whereas MMVT was most prevalent after PCI (Figure 1).

Patients with VA were more likely than patients without VA to have had a history of previous MI, more likely to have been using beta-blockers than patients without VA, and more VA patients smoked than patients without VA (Table 1). Also, patients with VA were more likely than patients without VA to have received cardiopulmonary resuscitation before arrival and mechanical chest compressions with the Lund University Cardiopulmonary Assist System. The 2 groups (with or without VA) did not differ as far as the right coronary artery, left anterior descending artery, or left circumflex artery as the infarct-related artery.

**Table 1:** Clinical characteristics

	Any VA (n= 120)	No VA (n = 2735)	Missing (%)	*P* value
Age, y	66.2 ± 10.7	66.8 ± 11.1	0.2	.527
Male	91 (76.5)	1975 (72.2)	0.2	.324
BMI, kg/m^2^	27.7 ± 4.2	27.3 ± 5.4	13.5	.360
Smoker	43 (35.8)	760 (28.7)	3.4	.036
Medical history				
Diabetes	18 (15.0)	379 (13.9)	0.5	.682
Hypertension	59 (49.2)	1267 (46.7)	0.8	.425
Previous MI	22 (18.3)	328 (12.0)	1.5	.030
Previous PCI	17 (14.2)	310 (11.3)	0.2	.326
Previous CABG	4 (3.3)	68 (2.5)	0.2	.542
Previous stroke	4 (3.3)	85 (3.2)	1.2	.787
Renal failure	13 (10.8)	383 (14.4)	2.5	.323
Chronic heart failure	79 (3.0)	3 (2.6)	2.8	1.000
Medications at admission				
Beta-blockers	15 (12.5)	103 (3.8)	1.1	<.001
Lipid-lowering therapy	33 (27.5)	616 (22.8)	1.2	.192
Digoxin therapy	0 (0.0)	6 (0.2)	2.6	1.000
Time from symptom to PCI, h	2.6 (1.8-4.4)	3.2 (2.1-5.6)	1.8	.002
Infarct-related artery				
RCA	36 (30.3)	957 (35.0)	—	.548
LAD	50 (42.0)	1076 (39.3)	—	
LCx	17 (14.3)	359 (13.1)	—	
LM	1 (0.8)	16 (0.6)	—	
Graft	2 (1.7)	20 (1.7)	—	
CPR before arrival	33 (27.7)	1 (0.1)	1.8	<.001
LUCAS	3 (2.5)	2 (0.1)	0.2	.001
LVEF <40%	37 (33)	45.2 (18.3)	9.7	<.001

Values are mean ± SD, median (interquartile range), or n (%), unless otherwise indicated. The *t* test, Mann-Whitney test, chi-square test, or Fisher’s exact test or were used.

BMI = body mass index; CABG = coronary artery bypass grafting; LAD = left anterior descending artery; LCx = left circumflex artery; LM = left main artery; LUCAS = Lund University Cardiopulmonary Assist System; LVEF = left ventricular ejection fraction; MI = myocardial infarction; PCI = percutaneous coronary intervention; RCA = right coronary artery; VA = ventricular arrhythmia.

In total, 2544 patients had an optimal result of primary PCI, while 336 patients had an impaired flow after PCI. Of those 336 patients, 55 patients had Thrombolysis In Myocardial Infarction (TIMI) flow grade 0, 38 patients had TIMI flow grade 1, and 243 patients had TIMI flow grade 2 after PCI.

Of the 38 patients with in-hospital VA after PCI (without regard to the 48-hour cutoff), 13 (34%) patients had an impaired flow in the infarct-related artery (TIMI flow grade <3) after PCI, while this was the case in just 11.4% (n = 323 of 2843) of patients without arrhythmias after PCI (*P <* .001). Three (8%) of the 38 patients with VA after PCI had TIMI flow grade 0 to 1 in the infarct-related artery after PCI, as compared with 90 (11.4%) of 2843 patients without arrhythmias after PCI (*P =* .115). In log binomial regression, TIMI flow grade <3 was associated with VT or VF after PCI with relative risk 4.10 (95% confidence interval [CI] 2.11–7.98; *P <* .001) in a univariable analysis and after adjustment for age, sex, and previous MI with an relative risk of 4.54 (95% CI 2.30–8.94; *P <* .001).

### Prognostic impact of VA

The in-hospital mortality was 10 times higher in the VA group than in the no VA group (Supplementary Table). The occurrence of VA of any type was associated with increased risk of in-hospital mortality (odds ratio [OR] 7.39; 95% CI 3.68–14.83; *P <* .001) after adjustment on age, sex, and MI localization. By the end of follow-up, 13 (10.8%) of 120 VA patients died, compared with 77 (2.8%) of 2735 patients without VA (*P <* .001). Cardiovascular death occurred in 12 (10.0%) patients in the VA group and in 64 (2.3%) patients in the no VA group (*P <* .001). After discharge, 48 deaths occurred, 36 of which were cardiovascular.

Compared with patients with no VA, early VA of any type was associated with total mortality and cardiovascular mortality after adjustment for age, sex and localization of MI (Table 2). In patients discharged alive, early VA did not significantly influence either total mortality (HR 0.56; 95% CI 0.77–4.05; *P =* .656) or cardiovascular mortality (HR 0.74; 95% CI 0.10–5.37; *P =* .763).

**Table 2:** Results of Cox regression analysis of association between mortality and the presence and type of ventricular arrhythmias (N = 2855)

	Total mortality (n = 89		Cardiovascular mortality (n = 75)	
	HR (95% CI)	*P* value	HR (95% CI)	*P* value
Any VA vs no VA	3.59 (2.01–6.42)	<.001	3.82 (2.08–6.99)	.001
PMVT/VF vs no VA	4.24 (2.91–8.21)	<.001	4.57 (2.72–9.20)	.001
MMVT vs no VA	4.67 (1.14–19.11)	.032	5.58 (1.36–22.96)	.017
MMVT vs PMVT/VF	1.06 (0.23–5.00)	.937	1.21 (0.25–5.74)	.815

HRs were adjusted for age, sex, and MI localization.

CI = confidence interval; HR = hazard ratio; MI = myocardial infarction; MMVT = monomorphic ventricular tachycardia; PMVT = polymorphic ventricular tachycardia; VF = ventricular fibrillation; VA = ventricular arrhythmia.

**Figure 2:**
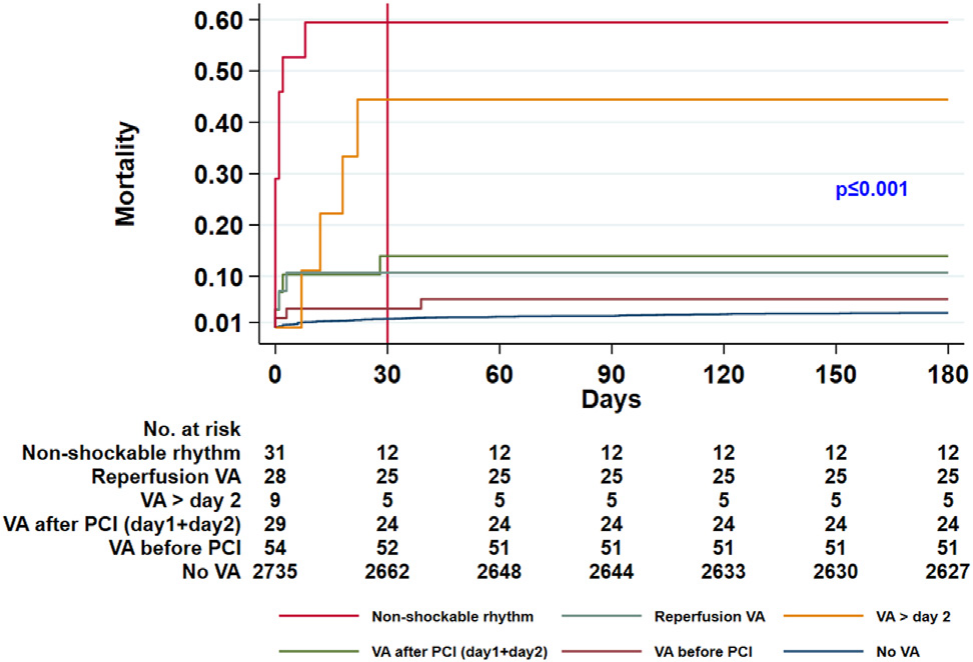
Kaplan-Meier failure curves: total mortality with regard to arrhythmia timing. PCI = percutaneous coronary intervention; VA = ventricular arrhythmia.

The timing of early VA was associated with the risk of death. Compared with patients without VA, VT or VF before PCI, VT or VF during reperfusion, and VT or VF after PCI were associated with an increased risk of mortality after adjustment for age, sex and MI localization (Table 3, Figure 2). VT or VF after PCI was associated with a further increased risk of death when compared with VT or VF before PCI (Table 3). Taking into account the fact that VT or VF after PCI was often associated with a nonoptimal PCI result, in order to assess the prognostic value of VT or VF after PCI, the Cox proportional model was adjusted not only for age, sex, and MI localization, but also for nonoptimal TIMI flow grade (<3) after PCI; the adjusted Cox proportional hazard of VT or VF after PCI for total mortality was 4.26 (95% CI 1.8–10.08) (*P =* .001).

**Table 3:** Results of Cox regression analysis: mortality prediction with regard to timing of ventricular arrhythmias (N = 2855)

	Total mortality (n = 89)		Cardiovascular mortality (n = 75)	
	HR (95% CI)	*P* value	HR (95% CI)	*P* value
VA before PCI vs no VA	3.14 (1.27–7.78)	.013	3.04 (1.10–8.36)	.03
Reperfusion VA vs no VA	1.52 (1.14–2.03)	.004	1.59 (1.19–2.13)	.002
VA after PCI vs no VA	2.61 (1.57–4.34)	<.001	2.87 (1.72–4.79)	<.001
VA after PCI vs VA before PCI	6.68 (2.90–15.41)	<.001	7.49 (3.12–17.99)	<.001

HRs were adjusted for age, sex, and MI localization.

CI = confidence interval; HR = hazard ratio; MI = myocardial infarction; PCI = percutaneous coronary intervention; VA = ventricular arrhythmia.

**Figure 3:**
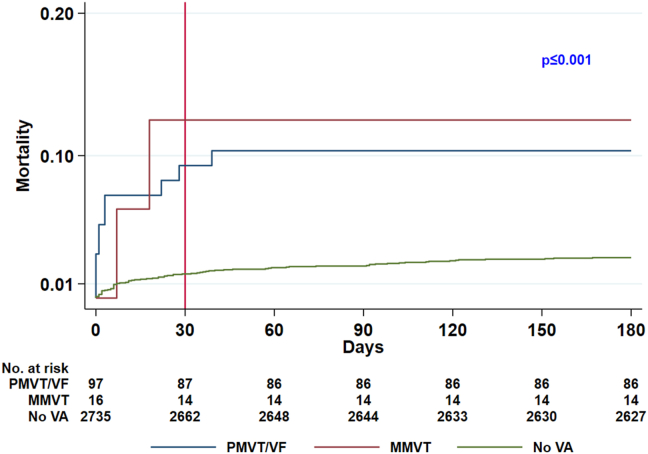
Kaplan-Meier failure curves: total mortality with regard to arrhythmia type (nonshockable rhythm excluded). MMVT = monomorphic ventricular tachycardia; PMVT = polymorphic ventricular tachycardia; VA = ventricular arrhythmia; VF = ventricular fibrillation.

VA type was not associated with the mortality risk. Two (12.5%) of 16 patients with MMVT and 10 (10.3%) of 97 patients with PMVT/VF died (*P =* .678). Of those, 2 (12.5%) and 9 (9.3%) patients, respectively (*P =* .654), died from cardiovascular causes. From the Cox model, neither total mortality nor cardiovascular mortality was associated with arrhythmia type (Table 2, Figure 3).

Among patients discharged alive, just 1 patient with PMVT/VF died by end of follow-up (from a cardiovascular cause), while no patients with MMVT died. We were not able to perform survival analysis with regard to the timing or the type of arrhythmia in patients discharged alive because of the small number of events.

## Discussion

In this prespecified analysis of VA from a registry-based randomized clinical trial based on nationwide data from Sweden, we report a relatively low prevalence of VAs associated with STEMI, 91% of which occurred during the first 24 hours and was strongly associated with in-hospital mortality while not affecting long-term prognosis.

In our material, almost half (50%) of all VA episodes occurred before PCI, while one-third occurred prior to hospital admission. These ratios are higher than previously reported in other studies: for example, in the Assessment of Pexelizumab in Acute Myocardial Infarction (APEX-AMI) study, precatheterization VA accounted for just 7.5% (25 of 329) of events; in the French registry of Acute ST-elevation or non-ST-elevation Myocardial Infarction study, the mean time from MI diagnosis to occurrence of VF was 1.8 days (95% CI 1.4–2.2). Our findings can be explained by the broad patient enrollment due to few exclusion criteria as part of a pragmatic registry-based trial. Our study did not exclude the most severe patient categories, such as patients who underwent prehospital resuscitation or arrived at the catheterization laboratory with ongoing mechanical chest compressions with the Lund University Cardiopulmonary Assist System. This may be explained by the difference between our patient population and patient populations selected in other randomized studies.

In our study, VA occurred after PCI in 31% of patients, which is in agreement with the APEX-AMI study (35%).^9^ VAs after PCI were associated with impaired TIMI flow grade in the infarct-related artery after PCI, which is in line with earlier findings of the Clopidogrel as Adjunctive Reperfusion Therapy-Thrombolysis in Myocardial Infarction 28 study on association of VA with impaired myocardial perfusion after fibrinolysis,^19^ and also in line with the findings of the APEX-AMI study, which reported the association between postprocedural TIMI flow grade <3 and postprocedural VA.^9^

Our data indicate that prehospital VA, if successfully cardioverted in a timely manner, in patients admitted for primary PCI is associated with the lowest in-hospital mortality (3.4%), even if compared with VA occurring in hospital before PCI (8%) and reperfusion arrhythmias (10.7%). The risk of mortality related to VA is associated with timing of early VAs, and VAs after PCI are associated with a significantly higher mortality risk than arrhythmias before PCI. These results are in agreement with APEX-AMI findings, which also reported an increased risk of death associated with VA after catheterization end.^9^

In a contemporary setting, we show that 180-day mortality associated with VA during the first 24 hours after symptom onset is determined by in-hospital mortality, with practically no events reported after discharge from the hospital.

In the contemporary STEMI cohorts, the incidence of early VA occurring after 24 hours (ie, during 24–48 hours from symptom onset) is extremely low and does not allow sufficient power to statistically assess their association with the long-term outcome. In our data, just 3 patients (0.1% of all included patients; 2.7% of patients with VA) experienced VA during the second day of STEMI, which is in line with previously reported data.^6^ This number of patients is too low to support the use of a 48-hour threshold for risk stratification with regard to the risk of death and implantable cardioverter defibrillator therapy.

The prevalence of MMVT in our study (0.5%) was lower than previously reported incidence (2.6%–3%)^13^^,^^20^; however, in our study, we only considered MMVT demanding cardioversion and not all sustained MMVT like previous publications did. Recently, we published a single-center study on a similar size study group treated 10 years earlier.^15^ The prevalence of MMVT in STEMI patients admitted for primary PCI was 1.5%. The clinical characteristics of patients and the time distribution of MMVT were quite similar in the 2 studies (the single-center study and the current study). PMVT/VF was most prevalent before PCI, while MMVT was most prevalent after PCI. The reason for this phenomenon is likely related to the time needed for an ischemic lesion, to develop, thus creating the arrhythmic substrate late in the course of STEMI. In the single-center study, we showed that early MMVT is associated with a higher risk of all-cause mortality than PMVT/VF. In the current study, both PMVT/VF and MMVT were associated with increased mortality as compared with patients with no VT or VF, but we could not state with certainty that MMVT is associated with greater mortality than PMVT/VF.

Thus, MMVT is a very rare finding in the acute STEMI population. It occurs mainly after PCI and is associated with a mortality hazard rate similar to VF.

### Limitations

Our study has several limitations. Because of the design of the main study (VALIDATE-SWEADHEART study), the follow-up period in our study was limited to 180 days. Due to VALIDATE-SWEADHEART study’s registry-based platform, specific information on implantable cardioverter-defibrillator implantation after MI, the use of wearable cardioverter-defibrillators, and sudden arrhythmic death information was not available for the current study.

The low incidence of VA and the low outcome event rates, especially in patients with VA within 24 to 48 hours of STEMI, and in the MMVT group did not allow sufficient power to statistically assess their association with the long-term outcome in these subgroups.

## Conclusion

Early VA is a marker of poor short-term outcomes in patients with STEMI, which does not affect long-term prognosis in patients successfully resuscitated and discharged from hospital. VA after PCI is associated with an increased risk of death compared with VA occurring before PCI. Monomorphic VT is a rare event during STEMI, thus precluding drawing conclusions with regard to its prognostic impact.

As the incidence of early VA after 24 hours of symptoms appears to be negligibly low, our results support the use of a 24-hour threshold defining VAs that do not affect long-term prognosis.

## Funding Sources

This study was supported by the Swedish Heart Lung Foundation (# 20180222 to MMD and #20200674 to PPG), by grants from the Swedish state under the agreement between the Swedish government and the county councils, the ALF agreement, and by donation funds at Skåne University Hospital.

## Disclosures

The authors have no conflicts to disclose.

## Authorship

All authors attest they meet the current ICMJE criteria for authorship.

## Patient Consent

Witnessed oral consent was obtained in all patients followed by providing written informed consent within 24 hours.

## Ethics Statement

The research reported in this paper was conducted in accordance with the guidelines outlined in the Declaration of Helsinki. The study was approved by the ethics committee at Lund University.
