# Supplementary material for: Prognostic value of early sustained ventricular arrhythmias in ST-segment elevation myocardial infarction treated by primary percutaneous coronary intervention: A substudy of VALIDATE-SWEDEHEART trial
Source: Heart Rhythm O2. 2022 Dec 22;4(3):200–6. doi: 10.1016/j.hroo.2022.12.008 (PMC10041082; doi:10.1016/j.hroo.2022.12.008)
Supplement: Supplementary Table [file mmc1.docx]

**Supplementary Table** Prevalence, time aspects and relation to in-hospital mortality of the shockable ventricular arrhythmias during STEMI (all patients, n=2,855)

|  | PMVT/VF  n=97 | MMVT  n=16 | Other shockable rhythm  n=7 | Non-shockable rhythm  n=31 | No VA  n=2735 |
| --- | --- | --- | --- | --- | --- |
| Before reperfusion | 49 | 3 | 2 |  |  |
| Reperfusion | 25 | 3 | - |  |  |
| After PCI | 23 | 10 | 5 |  |  |
| <24 h from symptom onset | 93 | 10 | 5 |  |  |
| 24-48h | 1 | 2 | - |  |  |
| >48 h | 3 | 4 | 2 |  |  |
| In-hospital death | 9 (9.2%) | 2 (12.5%) | 2 (28.5%) | 18 (58.0%) | 30 (1.0%) |
